# Metabolomic Insights into Human Arboviral Infections: Dengue, Chikungunya, and Zika Viruses

**DOI:** 10.3390/v11030225

**Published:** 2019-03-06

**Authors:** Nathaniel M. Byers, Amy C. Fleshman, Rushika Perera, Claudia R. Molins

**Affiliations:** 1 Division of Vector-Borne Diseases, Centers for Disease Control and Prevention, Fort Collins, CO 80521, USA; ngv8@cdc.gov (N.M.B.); muj3@cdc.gov (A.C.F.); 2 Arthropod-borne and Infectious Diseases Laboratory, Department of Microbiology, Immunology & Pathology, Colorado State University, Fort Collins, CO 80523-1692, USA; Rushika.Perera@colostate.edu

**Keywords:** arbovirus, metabolomics, metabolism, alphavirus, flavivirus, lipid

## Abstract

The global burden of arboviral diseases and the limited success in controlling them calls for innovative methods to understand arbovirus infections. Metabolomics has been applied to detect alterations in host physiology during infection. This approach relies on mass spectrometry or nuclear magnetic resonance spectroscopy to evaluate how perturbations in biological systems alter metabolic pathways, allowing for differentiation of closely related conditions. Because viruses heavily depend on host resources and pathways, they present unique challenges for characterizing metabolic changes. Here, we review the literature on metabolomics of arboviruses and focus on the interpretation of identified molecular features. Metabolomics has revealed biomarkers that differentiate disease states and outcomes, and has shown similarities in metabolic alterations caused by different viruses (e.g., lipid metabolism). Researchers investigating such metabolomic alterations aim to better understand host–virus dynamics, identify diagnostically useful molecular features, discern perturbed pathways for therapeutics, and guide further biochemical research. This review focuses on lessons derived from metabolomics studies on samples from arbovirus-infected humans.

## 1. Introduction

Metabolites are the downstream products of enzymes and cellular pathways and provide a window into the biochemical state of cells in varying conditions. A metabolite is a small molecule synthesized by catabolic or anabolic processes. Metabolomics is the systematic study of these small molecules, including amino acids, lipids, nucleotides, sugars, hormones and vitamins; virtually all organic compounds found in an organism except DNA, RNA, and proteins [1,2]. Metabolomics analyses can be performed on any biofluid, as well as on tissues or even breath [1,2,3].

Screening metabolite abundances in the blood of newborns has allowed clinicians to effectively ameliorate potentially devastating effects of inborn errors of metabolism since the 1960s by reducing the time to diagnose and treat these disorders that occur in thousands of infants per year [4,5]. By applying targeted mass spectrometry (MS) techniques, 49 inborn errors of metabolism can be identified at birth [6]. This highly successful program clearly demonstrates the utility of detecting abnormalities in the concentrations of small molecules, and that metabolomics data can transition into practical diagnostic tests. Like inborn errors of metabolism, arboviruses deplete particular small molecules during infection and cause increased levels of others- often inflammation mediators. By elucidating metabolic changes in diseased states, metabolomics can provide insights into therapeutic molecules that could inhibit virus multiplication or lessen symptoms of viral diseases. Additionally, changes in metabolite abundances can be exploited as prognostic and diagnostic tools for arboviral diseases.

### 1.1. Arboviruses

Arboviruses are, by definition, transmitted to vertebrate hosts via arthropods, which include mosquitoes, ticks, or fleas. They cycle between vertebrate and invertebrate hosts to survive, and in general are not effectively passed horizontally in one host type [7,8]. This review focuses on arboviruses in the *Flaviviridae* and *Togaviridae* families, namely dengue viruses (DENVs), Zika virus (ZIKV), and chikungunya virus (CHIKV), as these are human arboviral infections investigated by metabolomics techniques to date. These viruses have overlapping geographic ranges and initially emerged in Africa before spreading to other continents [9,10,11,12]. Vectored by *Aedes* genus mosquitoes, these arboviruses collectively infect millions of people per year [9,10,11,12]. Since they can often cause similar clinical symptoms and numerous complications, rapid and accurate diagnostics for treatment and prognostication of arboviral infections are imperative [10,13]. Both flaviviruses and togaviruses are enveloped viruses with single-stranded, positive-sense RNA genomes of approximately 11–12 kilobases [14]. Genome replication occurs via viral RNA-dependent RNA polymerase in membrane-bound replication spherules connected to the cytoplasm, but protected from cellular defenses [15]. As a result, these viruses significantly alter the host metabolic environment for their replicative advantage. The host metabolic pathways manipulated by viruses depend on the lifecycle of the virus, the immune defenses activated, as well as the tissues and cell types infected [16,17]. Research in arbovirus metabolomics has identified membrane lipids induced by DENVs for replication, and has detected both host inflammatory and viral lipid metabolic alterations [18,19,20]. Comparing patients infected with DENVs to febrile patients that have illnesses not caused by DENVs identified metabolites that may improve the clinical diagnosis of DENVs [21,22]. Prognostic biosignatures that separate severe forms of dengue from milder forms have been developed [21,22,23,24]. Metabolomic studies have suggested that prescribing uric acid or dihomo-γ-linolenic acid (DGLA) may ameliorate dengue disease [24,25]. Another study linked metabolites to the symptoms of CHIKV infection [26]. Molecules during human ZIKV infection involved in autophagy and potentially neurological complications associated with the severe forms of this disease have also been identified by metabolomic techniques [27,28]. These studies demonstrate the real potential for metabolomics to elucidate host–virus interactions to aid in the development of antiviral and symptomatic treatments as well as prognostic or diagnostic tests.

### 1.2. Metabolomics

Metabolomics analyses of viruses present unique challenges since these pathogens lack an independent metabolism and must instead commandeer host resources [16,17,29]. Arboviruses induce strong host responses, and the sheer number of virally infected cells lends to detectably aberrant levels of metabolites. Metabolomics is a powerful tool for studying natural infections in situ, as it provides a means to investigate broad changes during human infection without the need for a model system. Metabolomics studies often aim to identify biomarkers, which are compounds correlated to a particular disease state or condition. A biosignature, or metabolic signature, is a set of biomarkers that distinguishes a particular disease from the healthy state or related diseases.

**Figure 1:**
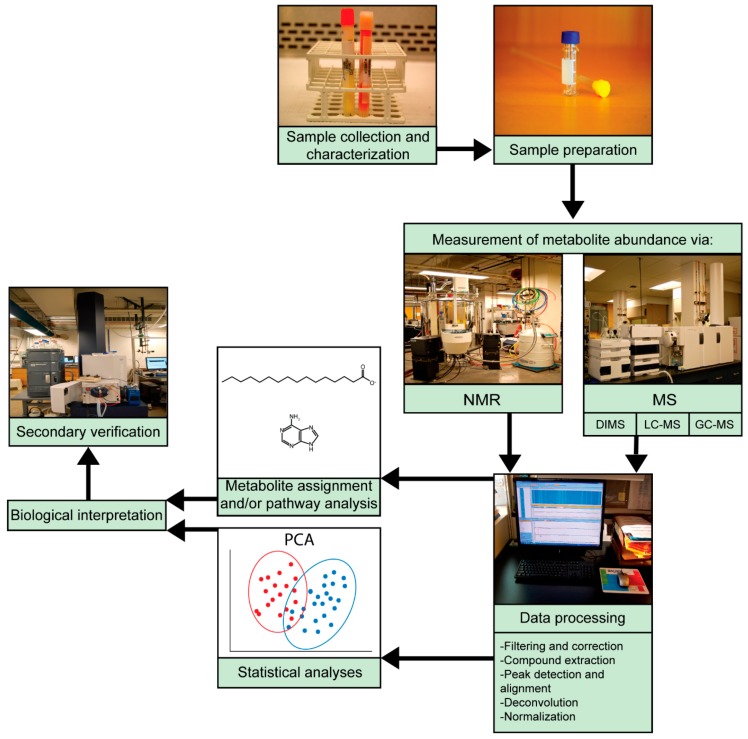
Workflow for metabolomic studies. Samples used for metabolomics analyses, most commonly serum or urine, should be carefully collected, stored, and characterized to confirm infection status and group membership. Metabolites are then quenched and extracted for nuclear magnetic resonance (NMR) or mass spectrometry (MS) analyses. Common MS techniques include direct-injection mass spectrometry (DIMS), liquid chromatography–mass spectrometry (LC-MS) and gas chromatography–mass spectrometry (GC-MS). Analytical methods generate data that must be aligned, extracted, corrected, and filtered to obtain molecular features. Metabolite identification and pathway analyses provide insight into the pathways perturbed during a disease state. Statistical analyses, such as principal component analysis (PCA) and partial least squares discriminate analysis (PLS-DA), differentiate and further classify samples and identify potential biomarker candidates. Once pathways and potential biomarkers have been elucidated, secondary verifications, such as targeted analyses, are often performed to confirm and further investigate biological connections, modes of action, or therapeutic potential of identified biomarkers.

Depending on the motives and end goals, metabolomics studies typically follow the same general workflow and can be divided into two main categories: untargeted studies and targeted studies (Figure 1). Untargeted studies are discovery-based and seek a broad, comprehensive picture of metabolites present in a system at a given time, including both identified metabolites and unknowns. In contrast, targeted studies aim to validate and quantify specific pre-defined metabolites, thus requiring more a priori knowledge [1]. Careful experimental design is required for both targeted and untargeted studies. Sample preparation prior to analysis varies by sample type and requires forethought to effectively halt enzymatic activity, prevent loss or degradation of metabolites, and optimally extract metabolites. Procedures for extracting metabolites have been comprehensively detailed elsewhere [30,31,32,33].

Sample preparation and extraction methods depend on the choice of analytical method. For example, lipidomics, a subtype of metabolomics focusing on lipid species, often requires specialized extraction and analytical techniques. Metabolites are most often analyzed using nuclear magnetic resonance (NMR) spectroscopy or MS-based techniques. NMR spectroscopy, based on exciting transitions between the spin states of magnetic nuclei [34], is rapid, quantitative, and highly reproducible in a variety of systems [35,36,37]. Additional advantages of NMR spectroscopy include minimal sample preparation, non-destructive methods, and the ability to perform comprehensive metabolite detection with a single measurement [35]. Despite many advantages, NMR spectroscopy has several limitations, namely, its low sensitivity and selectivity compared to MS-based methods [38], as well as a limited capacity for targeted approaches with current techniques [35]. Proton NMR (^1^H NMR) is one of the most common NMR spectroscopic methods due to short analytical time and relatively high sensitivity compared to other NMR methods [39].

MS is a common approach for both targeted and untargeted studies, boasting higher sensitivity and selectivity, as well as the ability to detect more metabolites in a single run than NMR spectroscopy [35]. Direct-injection (also termed infusion) MS (DIMS) is high-throughput as samples are directly injected into a mass spectrometer, reducing analysis time and bias [40]. Limitations of DIMS include ion suppression and residue formation within the instrument, though additional MS assays can overcome these limitations [41,42]. To improve resolution, MS is often combined with a chromatographic separation method such as liquid chromatography (LC) or gas chromatography (GC) to separate mixture components and simplify metabolite identification [38]. LC-MS and GC-MS generally require more laborious sample preparation than DIMS or NMR and include adaptations for compounds that are difficult to ionize [38]. GC-MS can only analyze volatile compounds and often requires derivatization of metabolites [43]. LC-MS is able to separate and detect a wide variety of molecules and is thus generally well-suited to global analyses of biological molecules. The most common LC-MS method is reversed-phase LC-MS (RPLC-MS), which is adept at separating most semi-polar compounds, but is not suitable for highly polar or ionic molecules [35,44]. However, techniques such as hydrophilic interaction liquid chromatography (HILIC) can increase sensitivity for analyzing polar metabolites [45]. Targeted experiments can include multiple reaction monitoring (MRM), which detects and quantifies analytes of a specific mass [46] and incorporates standard curves to quantify metabolites and confirm identities through comparison to standards. Detailed targeted methods have been previously reviewed [1,47,48].

Following data acquisition, spectral data are pre-processed according to the analytical method, and molecular features are then extracted and identified. Statistical analyses of metabolomics data are performed to assess differences between sample groups, identify differential metabolites, and classify samples based on these metabolites. Statistical analyses typically include both unsupervised approaches such as clustering analyses and principal component analysis (PCA), which seek to understand variation and trends within the data, and supervised approaches such as partial least squares discriminant analyses (PLS-DA), which can identify biomarkers and predict or classify sample group membership [49]. Once putative biomarkers have been identified and statistical and significance analyses have been performed, functional interpretations elucidate biological connections between metabolites and identify perturbed metabolic pathways. Pathway analysis tools include MetaboAnalyst [50], Mummichog [51], Kyoto Encyclopedia of Genes and Genomes (KEGG) [52], and MetaCyc [53], among others [54]. Efforts to inform biological understanding from metabolomics data require molecular features to be identified as metabolites by probing existing databases such as METLIN [55], the Human Metabolome Database (HMDB) [56,57,58], MassBank [59], and LIPID MAPS [60]. Identifying perturbed pathways and specific metabolites can be challenging, particularly when performing untargeted analyses, so additional verification, often by tandem MS, is necessary. Biological explanations for findings lend additional confidence to metabolomics results. Additional software tools for processing and analyzing metabolomics data are detailed elsewhere [61,62,63,64,65,66].

While no single analytical platform or methodology is able to detect and quantify all the components of a sample, some platforms are better suited to certain metabolites, sample types, or desired study outcomes. Regardless of the approach chosen, it is crucial to minimize variation at each step of the workflow. Establishing and following standard operating procedures reduces bias in sample preparation and processing [67]. Variation in biological samples is widespread, and while some variables such as patient age and gender produce real and interesting differences in metabolomics data, some variables, such as patient sampling time, diet, and medications, produce irrelevant artifacts that can heavily affect data and skew results. To account for the latter variation, it is necessary to “normalize” the data, effectively removing unnecessary biases while preserving authentic biological variation. Although there is no standard normalization method, most methods fall into two broad categories: normalization via the inclusion of quality control and internal and/or external standards [68] that are run and processed along with other samples, and normalization by data-scaling and statistical methods [69,70,71,72,73]. Additionally, metabolomic studies normalize data by comparing the group of interest to a control group. Groups must have sufficient sample numbers to compensate for individual variation and discover metabolites that legitimately separate groups.

Standardization in reporting results is another factor critical to accurate interpretation of metabolomics data. The Metabolomics Standards Initiative (MSI), created to guide analytical techniques and reporting practices, is an excellent step toward standardization of metabolomics methods [74]. However, the MSI is not comprehensive and will require additional updates as the field progresses [74].

Interpretation of metabolomics data requires, as with all steps of the workflow, acute discretion and attention to detail. Optimally, metabolite identities are confirmed, often by tandem MS (MS/MS) [75]. Orthogonal techniques should be used to validate metabolite abundances. Many of the same metabolites are perturbed for unrelated diseases due to the common host pathways altered by different pathogens. However, levels of overlapping metabolites often differ during the course of infection and require deeper investigation to understand infection processes and their progression.

## 2. DENV

DENVs are among the most clinically important arboviruses as they infect 390 million people per year and cause about 9000 deaths worldwide [12,76]. DENVs have four serotypes, numbered one to four. Infection with one serotype does not provide immunity to infection with other serotypes. Diseases caused by these viruses range from asymptomatic to lethal infection. Dengue fever (DF) is a five-to-seven-day acute febrile illness characterized by headaches as well as joint, muscle, and bone pain [77]. Most patients recover from DF. Subsequent infection with a second serotype can result in a more severe form of disease, Dengue Hemorrhagic Fever (DHF), which is characterized by plasma leakage into pleural and abdominal cavities [77]. Though classified as different diseases, DHF mimics the symptoms of DF early in infection [77]. The worst subset of DHF cases is termed Dengue Shock Syndrome (DSS), in which the patient develops hemorrhage and profound hypotension. Patients with grade IV DHF/DSS have undetectable blood pressure and pulse and are at serious risk of dying [77]. These disease categories are based on the World Health Organization’s (WHO) 1997 criteria, which were refined in 2011 [77,78]. While DHF can be caused by primary infection, DHF and DSS are principally due to antibody-dependent enhancement of viral entry during secondary infection [14,77].

### 2.1. Infection with DENVs Alters Cell Membrane Composition (Glycerophospholipids and Sphingolipids)

**Figure 2:**
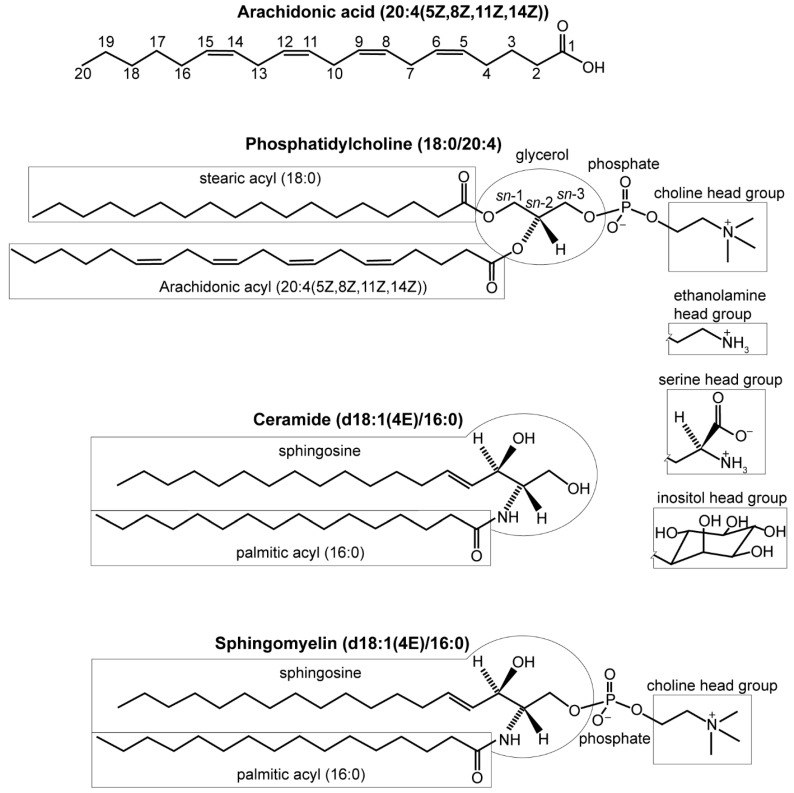
Lipid structures. LIPID MAPS naming conventions indicate “carbon atoms”: “double bonds” (location and conformation of double bonds) [80]. Phosphatidylcholine (PC) is composed of two lipid tails esterified to glycerol 3-phosphate with a choline head group. Glycerol’s stereospecific carbons are numbered *sn*-1, *sn*-2, and *sn*-3 [81]. Phosphatidylethanolamine (PE), phosphatidylserine (PS), and phosphatidylinositol (PI) have the same structure, but with alternative head groups. Ceramide (Cer) contains a sphingosine backbone, and a fatty acyl group. Sphingomyelin is Cer with a phosphocholine or phosphoethanolamine head group. The “d” in the name indicates that these have 1,3-dihydroxy sphingosine backbones.

DENVs induce alterations in many host lipids, including fatty acids, glycerolipids, glycerophospholipids, and sphingolipids. A fatty acid is a carboxylic acid with an aliphatic carbon chain that is typically unbranched and 4–28 carbons long [79]. The carbon chains can be unsaturated (containing double bonds) or saturated (no double bonds). Fatty acids can exist free or esterified into larger molecules (Figure 2).

Phosphatidylcholine (PC), phosphatidylethanolamine (PE), phosphatidylinositol (PI), phosphatidylserine (PS), and sphingomyelin (SM) are major components of cell membranes (abbreviations listed in Table 1) [82]. PC, PE, PI, and PS are glycerophospholipids with a 3-phosphoglycerol backbone esterified to a pair of fatty acyls with the *sn*-2 site enriched in unsaturated fatty acyls [81,83,84]. Within these classes of molecules, species vary by the length and saturation of their fatty acyl tails. Lipid tails can be ether-linked instead of the prototypic ester linkage. Lysophospholipids (LPL) are variants of glycerophospholipids that have one fatty acyl chain hydrolyzed, often by phospholipase A2, so only one remains.

Metabolomics studies on dengue patients revealed alterations in glycerophospholipids and sphingolipids (Table 2). The first metabolomics study on human sera from patients infected with DENV1-3 identified decreased levels of PC, lysophosphatidylcholines (LPC), and lysophosphatidylethanolamines (LPE) and increased levels of SM(d18:1/18:1(9Z)) relative to healthy control patients [20]. Increased SM levels correlated with decreased lymphocyte counts and LPE levels positively correlated to platelet counts. The timing of these changes connected elevated SM with the early host response while increased LPE levels correlated to later responses, and thus these lipids may have potential as prognostic biomarkers [20]. Khedr et al. [85] also found four of the same LPCs similarly decreased during infection with DENVs in adult sera relative to healthy controls. LPCs have also been shown to have prognostic potential in a cohort of children in Nicaragua with DENV2 [22]. In this study, patients who had DHF had higher levels of LPCs than non-dengue controls [22]. LPCs have been linked to increased endothelial permeability, a hallmark of DHF/DSS [77,86]. The conical shape of LPCs induces positive membrane curvature, which may be crucial for the functionality of the virion envelope or formation of the replication spherules, as both are highly curved [87]. Other work identified seven glycerophospholipids including PSs, PEs, and LPEs that predicted onset of DHF in adults in Singapore [23]. Of these three classes, only the LPEs were consistently decreased in patients with DHF; PSs and PEs were increased or decreased according to their particular fatty acyl composition. These studies demonstrated that glycerophospholipids have considerable potential as prognostic biomarkers predicting the likelihood that a patient has DHF.

Glycerophospholipids can also differentiate viral diseases. Khedr et al. [85] used LC-MS/MS to quantify glycerophospholipids in sera from healthy controls and patients diagnosed with DF, hepatitis B, or hepatitis C. The authors attributed the decreased PIs and PE in DF patients compared to healthy controls to phospholipase A2 activation by DENVs, which would convert them to lysophosphatidylinositols (LPI) and LPE [85,91]. Indeed, virus infection increased the amount of LPI(16:0), a potential product of phospholipase A2 activity on the PIs. PSs and LPCs were also less abundant during DF than in healthy controls [85]. Levels of several PCs, PE(38:4), and PS(36:2) were higher and levels of LPCs were lower in DF patients than those of hepatitis B or hepatitis C patients, thereby differentiating these viral diseases [85].

Another group, Melo et al. [89], found seven PCs that were elevated in the sera of DENV4-infected patients in Brazil (within four days of symptom onset) relative to healthy controls using DIMS and MS/MS. Because some of the PCs had ether-linked fatty acyls, the authors speculated that three of the PCs are precursors to the platelet activation factor, which has been linked to thrombocytopenia and vascular permeability during DENV2 infection [89,92]. Alternatively, the PCs may contribute to the formation of the viral spherule membranes [89].

Cui et al. [93] used metabolomics to validate a humanized mouse model for DENV2 infection (Table 3). They found that SMs were elevated in the sera within seven days of DENV2 inoculation, while glycerophospholipids including PE, LPE, and PC mostly decreased, consistent with previous results in human sera from the same group [20,93]. This study emphasized the value of metabolomics to broadly evaluate a model system and compare it to human infection [93].

### 2.2. Fatty Acid Levels Are Influenced by Infection with DENVs

**Figure 3:**
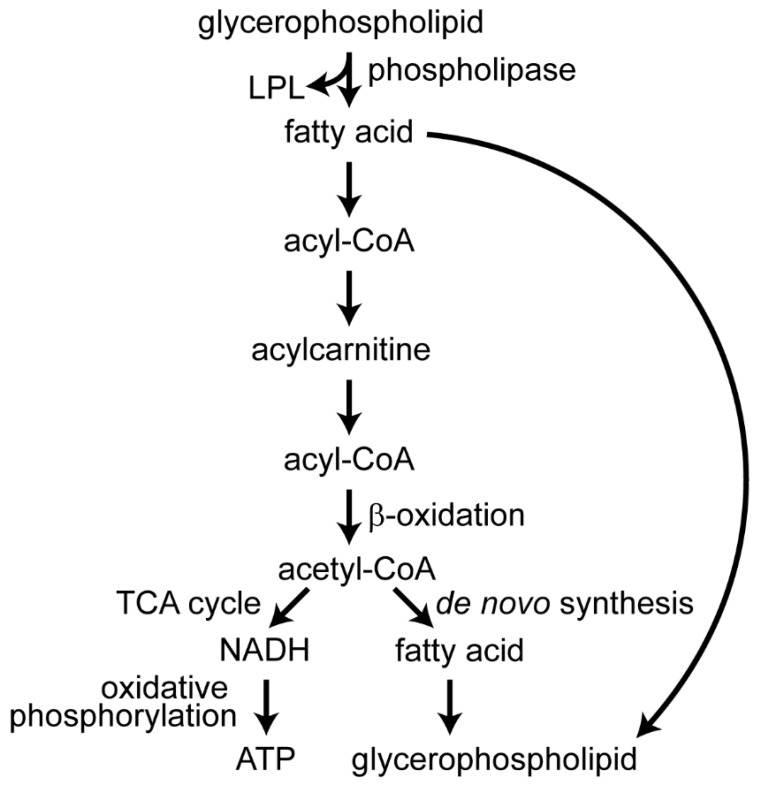
Fatty acid metabolism. Fatty acids are freed from glycerophospholipids by phospholipases producing lysophospholipids (LPL). Fatty acids are then conjugated to coenzyme A (CoA). The fatty acyl is bonded to carnitine for transport to the mitochondrial matrix, where they can undergo β-oxidation generating acetyl-CoA. The acetyl-CoA can enter the tricarboxylic acid (TCA) cycle to generate NADH which will produce adenosine triphosphate (ATP) during oxidative phosphorylation, or the acetyl-CoA can be used to build new fatty acids and glycerophospholipids. The fatty acids can also be used to remodel glycerophospholipids in the Lands’ cycle without being degraded [98].

Glycerophospholipids provide a reservoir for unsaturated fatty acids and the Lands’ cycle controls the balance of free fatty acids and their incorporation in glycerophospholipids [98]. Phospholipase A2 cleaves the fatty acyl group from the *sn*-2 carbon of glycerophospholipids, freeing the fatty acid that is often unsaturated, such as arachidonic acid (AA), docosahexaenoic acid (DHA), and eicosapentaenoic acid (EPA) (Figure 3) [81,83,84]. Unsaturated fatty acyls incorporated into glycerophospholipids influence membrane properties [83].

In the serum of people with DF in Singapore, AA, a pro-inflammatory fatty acid, was elevated during the febrile and defervescent phases [20]. AA comprises up to 25% of the fatty acyls in cell glycerophospholipids [81]. Eicosanoids produced from AA can be both pro- and anti-inflammatory and mediate the overall response [84,99]. Hence, AA may contribute to DF symptoms and limiting viral multiplication. In contrast, the increases in anti-inflammatory molecules during the febrile phase of DF, including DHA, inosine, and cortisol, are likely the body mitigating the inflammation, though these metabolites may aid viral persistence [20]. This study also detected elevated α-linolenic acid (ALA), a dietary fatty acid and a precursor to DHA, in human sera during infection with DENV1-3 [20]. Voge et al. [22] found that AA, DHA and ALA could predict DHF, as levels were higher in patients with DHF than those with DF in their cohort of children in Nicaragua. Both Cui et al. [20,25] and Voge et al. [22] found increased DHA in adult DF compared to in healthy controls, but substantially decreased DHA in adult DHF/DSS compared to in DF, a markedly different result than the increase in DHA found in children in Nicaragua with DHF. Cui et al. [93] also found increased AA, DHA, and other fatty acids in the serum of DENV2-infected humanized mice. These studies link fatty acid metabolism to the symptoms induced by DENVs, implying this pathway may be a potential therapeutic target.

### 2.3. Infection with DENVs Alters Glycerolipid Utilization

Glycerolipids can have one, two, or three fatty acyl groups esterified to a glycerol backbone and are termed mono-, di- and triacylglycerols (or triglycerides). Glycerolipid usage is altered in humans during infection with DENVs [24,88,89]. Increased levels of four triacylglycerols differentiated DENV4-infected patients in Brazil during febrile illness from healthy controls [89]. These glycerolipids may be mobilized to provide energy for viral replication or acetyl-coenzyme A (acetyl-CoA) for de novo fatty acid biosynthesis [89]. To study the alterations in the composition of fatty acyl-containing molecules during DF, Khedr et al. [88] used targeted GC-MS lipidomics to analyze esterified fatty acyls in the blood. In serum, esterified fatty acyls are mostly found in cholesterol esters, triacylglycerols, and glycerophospholipids [100]. The blood of patients with DF had reduced abundances of most fatty acyls targeted, compared to blood of healthy controls, consistent with reduced serum glycerophospholipids noted during infection by DENVs in Cui et al. [20] and Voge et al. [22,88]. The fatty acyls assayed by Khedr et al. [88] were chemically derived from lipids in whole blood, unlike the free fatty acids that Cui et al. [20] and Voge et al. [22] assayed in human sera. This work was extended by Villamor et al. [24], who assayed levels of most of the same fatty acids chemically derived from larger lipid molecules using targeted GC-MS metabolomics to prognostically differentiate DHF/DSS from DF. The sera used were collected from Columbian dengue patients within 96 hours of the onset of fever. Villamor et al. [24] found that elevated DHA and AA levels and lower levels of DGLA and pentadecanoic acid relative to total fatty acids indicated the patient likely had DHF/DSS rather than DF. Because it inhibits inflammatory eicosanoid biosynthesis, Villamor et al. [24] proposed DGLA as a potential therapeutic against DENVs. This work helped quantify changes in polyunsaturated fatty acyls that may be responsible for mediating inflammatory responses during severe dengue.

### 2.4. Metabolites Associated with β-Oxidation Are Perturbed during Infection with DENVs

Fatty acids from both glycerophospholipids and glycerolipids can undergo β-oxidation to produce acetyl-CoA, which can be used for de novo fatty acid synthesis, or they can enter the tricarboxylic acid (TCA) cycle to generate reduced nicotinamide adenine dinucleotide (NADH), reduced flavin adenine dinucleotide (FADH_2_), and guanosine triphosphate (GTP) [101]. The NADH undergoes oxidative phosphorylation to generate adenosine triphosphate (ATP). Acylcarnitines are intermediates for transporting fatty acids for oxidation in the mitochondria, and have one fatty acyl group esterified to carnitine [101].

Human sera contained elevated acylcarnitine levels during the febrile and defervescent phases of DF [20]. Cui et al. [20] suggested this aberrant β-oxidation indicated liver dysfunction. Acylcarnitine levels were lower in DHF patients than in DF patients, which may suggest differential perturbation of β-oxidation in severe versus mild dengue [20,25]. Bile acids were elevated during the defervescent phase of DF relative to healthy controls, implying liver damage, and were five times higher in DHF patients than in DF patients [20,25,102]. Elevated bile acids were significantly correlated with elevated aspartate transaminase and alanine transaminase levels in the blood, consistent with liver swelling and damage in DF and potential liver failure in DHF [25,103]. Similarly, bile acids, liver transaminases, and metabolites involved in fatty acid β-oxidation were elevated in the sera of DENV2-infected humanized mice, indicating liver damage [93].

Other researchers found other molecules that suggest aberrant β-oxidation and liver function. In a study in Brazil using ^1^H NMR to assay sera of patients with DF or DHF, very-low-density lipoprotein/low-density lipoprotein (VLDL/LDL) levels were lower in all dengue disease states than during non-dengue disease, perhaps due to liver dysfunction and altered β-oxidation [21]. Supporting this hypothesis, increased acetate levels, which correlate to liver disease [104], occurred in DHF patients relative to non-dengue control patients [21]. Free acetate, principally produced by intestinal flora, is bonded to CoA in the liver by acetyl-CoA synthetase as an alternative to pyruvate for the TCA cycle or fatty acid synthesis. In the urine of men with DF five to seven days post symptom onset, acetoacetate, a ketone body, was increased [21]. This elevated level of acetoacetate was likely produced in the liver from β-oxidation, suggesting that acetoacetate is being used to transport energy throughout the body in place of glucose, as occurs during fasting [105]. The increase in urine acetoacetate suggests dysregulation of fundamental energy storage and usage pathways during DENV3 infection.

These studies demonstrated perturbed β-oxidation in dengue disease, which implies liver dysfunction. It has been noted in the literature that infection with DENVs can cause liver swelling and elevated levels of liver transaminases [77,78], but a number of liver-related metabolites found by different studies imply more dysfunction than is generally recognized. In rats, Chang et al. (2017) demonstrated that liver fibrosis altered the TCA cycle as well as tryptophan, glycerophospholipid, and sphingolipid metabolism [106]. These same pathways were also altered in humans and mice infected by DENVs, suggesting that DENVs induce liver damage [20,21,22,23,25,27,85,90,93,95,96]. Levels of bile acids, acylcarnitines, and acetate were also increased in the sera of humans infected with DENVs, similarly suggesting liver damage [20,21,25]. Confirming the link between liver damage and certain metabolites, Cui et al. [25,93] found elevated liver transaminases correlated with elevated bile acids. Acceptance that DENVs cause liver damage is gaining traction [107,108], though the sensitivity of metabolomic findings suggests earlier mild damage than previously appreciated.

### 2.5. Amino Acid Usage Is Redistributed by Infection with DENVs

Amino acids do more than comprise the building blocks of proteins; they are also important sources of carbon and nitrogen and are precursors to many other metabolites, including serotonin and NAD^+^ [101]. Serotonin is important both as a neurotransmitter and to enhance blood clotting, while NAD^+^ is required for oxidative phosphorylation, the primary pathway for generating ATP [101,109]. Catabolism of amino acids can generate ATP [101].

Though 95% of tryptophan is converted to NAD^+^ via the kynurenine pathway, it can also be converted to serotonin or catabolized to pyruvate and acetyl-CoA to enter the TCA cycle [101,110,111]. Levels of tryptophan and kynurenine were altered during infection with DENVs in cell culture models and human sera, suggesting that these viruses alter inflammatory responses, as this pathway impacts and is influenced by inflammation [20,23,95,96,111]. DENV3 infection increased tryptophan levels and DENV4 altered kynurenine levels in the media of infected human cells [95]. Although not highlighted by Fontaine et al. [96], the supplemental table from their publication indicates that kynurenine had the largest fold increase of any metabolite detected at 24 hours post infection (hpi) (100-fold) and 48 hpi (49-fold), during DENV2 infection of human primary cells, whereas levels of tryptophan were significantly decreased. Based on their MS analysis of human sera, Cui et al. [23] proposed that products of tryptophan metabolism were prognostic for DHF. The authors compared levels of various metabolites during the febrile phase of infection with DENVs to cytokine assays and medical records to differentiate patients that developed DHF from those that resolved DF [23]. DF patients had lower serotonin and higher kynurenine levels than healthy controls, while DHF patients had even lower levels of serotonin and higher levels of kynurenine than DF patients within 96 hours of fever onset [23]. The elevated levels of serotonin in DHF patients compared to DF patients persisted through the defervescence/critical phase five to seven days post fever onset [25]. Plasma serotonin, which is a separate pool from that of the central nervous system, resides in granules in platelets and enhances platelet aggregation when released [109]. Thus, Cui et al. [23] postulated that the reduction in serotonin is due to the reduction in platelets during the thrombocytopenia characteristic of DHF. Reduced platelet counts and plasma serotonin levels can be detected before patients become critical [23,77]. When both serotonin and interferon-γ levels are used for a prognostic test, Cui et al. [23] achieved an early predictive power of 77.8% sensitivity and 95.8% specificity in differentiating DHF from DF on the samples utilized in this study. Though the roles of tryptophan metabolism in dengue disease are not yet well elucidated, metabolomic approaches indicate that these pathways may play important roles in virus multiplication and pathogenesis, which can be exploited to develop a prognostic assay.

Proline and glutamine are also potentially prognostic amino acids. Voge et al. [22] identified proline to predict progression to DHF during the febrile phase of DENV-2 infection in children in Nicaragua. Glutamine levels are influenced in cells and human sera by infection by DENVs [21,26,96]. Glutamine is the most abundant free amino acid in plasma [112]. In addition to its obvious role in protein synthesis, it is an important source of carbon, nitrogen, and energy for lipid synthesis, the TCA cycle, and other anabolic processes [112]. Elevated levels of glutamine have been linked to inflammatory cytokine production, a fundamental aspect of DHF/DSS responsible for increased disease severity [77,113]. Because of its role in numerous metabolic processes, glutamine usage is likely altered by viruses to optimize the balance of different glutamine metabolites useful for viral replication. Glutamine and glutamate levels were higher in DENV2-infected primary human cells than in mock infected at all time points [96]. Removing exogenous glutamine reduced infectious virus by 60% [96]. Glutamine may be replenishing the TCA cycle’s intermediates during infection [96]. In human sera, glutamine levels decreased with increased dengue severity, indicating that it may be catabolized for energy or carbon metabolism during viral infection [21].

In human sera five to seven days post fever onset, levels of phenylalanine were higher and uric acid levels were lower in DHF than in DF, possibly from higher oxidative damage in DHF [25]. Nitric oxide signaling, a part of oxidative stress that helps activate monocytes, may be a host immune response attempting to restrict replication of DENVs, but also is correlated to reduced platelet aggregation and DSS [114]. Conversion of phenylalanine to tyrosine relies on the cofactor tetrahydrobiopterin, which is oxidized during oxidative stress, causing an accumulation of phenylalanine. Decreased levels of uric acid, a major antioxidant in the blood, may allow for the oxidative damage previously reported during infection with DENVs [114]. Hence, Cui et al. [25] proposed that administering uric acid would decrease oxidative damage from viral infection and alleviate DHF, a valuable potential therapeutic technique. Shahfiza et al. [90] noted increased levels of 4-hydroxyphenylpyruvate, a downstream product of phenylalanine and tyrosine metabolism, in the urine of men with DF on the fifth through seventh days post symptom onset compared to healthy controls. This suggests that tyrosine was processed to 4-hydroxyphenylpyruvate. DF patients also had an increase in valerylglycine in their urine [90]. While little is known about valerylglycine metabolism, increases in other urine acylglycines indicate dysregulation of amino acid or fatty acid metabolism [115]. As with humans, Cui et al. [93] also found molecules related to phenylalanine, tryptophan, lysine, arginine and proline metabolic pathways elevated in the sera of DENV2-infected humanized mice within seven days post infection (dpi).

Many amino acids and their metabolites have been implicated in infection with DENVs. Several, including proline, serotonin, and kynurenine, may be useful prognostics for differentiating patients likely to decline with DHF and who will need extra medical intervention from those who will recover from DF [22,23].

### 2.6. DENVs Affect Additional Pathways

Alterations induced by infection with DENVs have been observed in many other metabolic pathways related to energy transfer and biosynthetic anabolism, including nucleotide and vitamin D metabolism, the TCA cycle, and glycolysis. Cui et al. [20,93] demonstrated in human sera and humanized mice sera that the metabolic disturbances induced by DENVs are greatest early in infection, and slowly return to healthy levels over time, with defervescent samples falling in between healthy and febrile samples by PCA. The patterns of these alterations mirror the course of infection, indicating that DENVs do not induce widespread, long-lasting perturbations in host metabolism.

Several studies evaluated the ability of metabolomics to differentiate DENV serotypes. In carefully controlled cell culture experiments, all four serotypes were differentiated [94,95]. Cui et al. [20] were not able to identify metabolites in human sera that differentiated DENV1 and DENV3 [20]; perhaps because these are the most closely related serotypes, or because the techniques applied lacked the sensitivity required for this task [9,14,116]. However, Voge et al. [22] found 25 metabolites that differentiated DENV1 and DENV2 serotypes in human sera, and Villamor et al. [24] found some fatty acyls that varied between the different serotypes during human infections.

Glycolysis is a fundamental process of cellular metabolism and was found to be altered by DENV2 in human foreskin fibroblasts by LC-MS and GC-MS on cell lysates by Fontaine et al. [96]. Levels of glucose 6-phosphate and fructose 6-phosphate, which are glycolysis intermediates, were significantly increased in infected cells at 24 and 48 hpi, whereas levels of downstream glycolysis metabolites, phosphoenolpyruvate and 3-phosphoglycerate, were elevated at 10 hpi, but decreased by 48 hpi when compared to levels in uninfected cells. These findings may suggest that elevated glycolysis produces downstream products during early infection and slows such that upstream intermediates accumulate during the later stages of infection. Inhibition studies determined that glycolysis is required for DENV2 replication, as withholding glucose from the medium or inhibiting glycolysis reduced virus replication about 100-fold [96]. Fontaine et al. [96] hypothesized that glycolysis is necessary for lipid biosynthesis induced by DENVs to form replication spherules or perhaps other replicative requirements, such as generating ATP. By utilizing glycolysis, DENVs could save fatty acids for membrane synthesis rather than use them for β-oxidation.

Shahfiza et al. [90] found that the TCA cycle, which is downstream of glycolysis, was perturbed by infection with DENVs. Levels of succinate and citrate were decreased in the urine of infected men on the fifth through seventh days post symptom onset compared to those in healthy controls, indicating that this fundamental pathway for fueling the body is perturbed by infection [90]. The authors suggested these and 11 other metabolites they identified as altered in the urine of men infected by DENVs are potential diagnostic molecules [90].

Other metabolites altered by infection with DENVs include vitamin D_3_, purines, and pyrimidines. Decreased levels of 1,25-dihydroxyvitamin D_3_, the biologically active version of vitamin D_3_, correlated to DHF/DSS compared to DF and non-dengue disease in the Nicaraguan cohort analyzed by Voge et al. [22]. Though principally associated with calcium regulation, vitamin D_3_ can also influence the vascular barrier and immunoregulation [117]. Hence, reduced 1,25-dihydroxyvitamin D_3_ production may be partially responsible for the symptoms of DHF/DSS [22]. Cui et al. [93] found purine and pyrimidines elevated in the sera of DENV-infected humanized mice within 7 dpi, perhaps to generate ribonucleotides for viral genome replication.

Over a dozen articles have illuminated the metabolic alterations caused by infection with DENVs in model systems and humans, implicating lipid and fatty acid changes, as well as changes in glycolysis, the TCA cycle, β-oxidation, amino acid metabolism, and many other affected pathways. These studies are valuable as they suggested metabolites for diagnostic and prognostic assays, proposed therapeutics such as uric acid and DGLA, and elucidated host–virus interactions [24,25,90].

## 3. CHIKV vs DENVs

CHIKV is an alphavirus in the *Togaviridae* family, but shares epidemiological and ecological features with the flaviviruses DENV1–4 and ZIKV, as they have overlapping geographic ranges and are transmitted by the same *Aedes* vectors [9]. Local transmission of CHIKV has been documented on five continents, and there is substantial risk for CHIKV to spread to Australia [118]. Chikungunya fever, which has affected millions of people over the last two decades, is an acute febrile illness with joint arthralgia that can persist for months or years following fever resolution [9]. Identifying metabolic features that distinguish CHIKV from other tropical diseases could substantially improve diagnoses for populations at risk of contracting this and clinically similar diseases.

To differentiate metabolic alterations induced by CHIKV from DENVs, Shrinet et al. [26] analyzed sera from patients in India infected with CHIKV, DENVs, or co-infected with both using ^1^H NMR (Table 4). Shrinet et al. [26] found alterations in glycine, serine, threonine, and galactose metabolism, as well as the TCA cycle, in CHIKV patients relative to healthy controls. Alterations in the TCA cycle are expected given CHIKV’s high energy requirements for rapid multiplication and generation of biosynthetic building blocks for lipids, proteins, and RNA. The authors found statistical correlation between joint damage and elevated hypoxanthine and 4-hydroxyphenylpyruvic acid levels during CHIKV infection, consistent with their reported involvement with arthritis [26,119,120]. This finding provides evidence for conserved molecular features between CHIKV-induced arthralgia and rheumatoid arthritis. As for CHIKV infection, glycine, serine, threonine, and galactose metabolism were also altered in patients infected with DENVs and in patients co-infected with CHIKV and DENVs versus healthy controls. Glutamine levels were noted to increase only in co-infections, which may be due to the statistical selection of metabolites, as other work has found glutamine levels altered by infection with DENVs alone [21,26,96]. Several biomarkers differentiated CHIKV infections from infections with DENVs, including sorbitol, 2-ketobutyric acid, and sarcosine [26]. Sorbitol was elevated in CHIKV patients relative to healthy controls, but was not detected in dengue patients, and was observed at an intermediate level in co-infected patients. Sorbitol is an intermediate in sugar metabolism, and therefore, virus-specific changes suggest that CHIKV and DENVs cause different alterations in carbon metabolism.

## 4. ZIKV

ZIKV is an emerging flavivirus that has recently expanded from Southeast Asia to the Americas. With only 13 naturally acquired cases reported between the discovery of ZIKV in humans in 1953 and an outbreak on Yap Islands in Micronesia in 2007, the recent expansion of ZIKV was surprising [13]. Also surprising was the discovery that ZIKV can be sexually transmitted, though it is primarily vectored by *Aedes* mosquitoes [7,125,126]. ZIKV usually causes no symptoms or only a mild febrile illness with rash, but can cause severe diseases such as microcephaly during fetal development or the paralytic autoimmune disorder, Guillain-Barre syndrome [126].

Melo et al. [27,28] investigated unique metabolites in human serum using DIMS. They selected metabolites that were detected in the serum of ZIKV-infected patients, but were not detected in non-ZIKV ill patients nor detected in healthy controls. They found increased levels of PI phosphates (PIPs), which are PI with an additional phosphate on the sugar moiety, and angiotensins. Both PIPs and angiotensins contribute to mTOR signaling to prevent autophagy. Like DENVs, ZIKV induces autophagy, presumably to increase β-oxidation and aid viral multiplication [127,128]. The hosts may increase angiotensins and PIPs to restrict autophagy and deprive ZIKV of the nutrients it would obtain from β-oxidation. However, ZIKV NS4A and NS4B proteins inhibit the mTOR signaling pathway that is stimulated by PIPs and angiotensins, thus allowing for autophagy and making this potential host defense ineffective [127]. Melo et al. [27,28] also identified an increase in ganglioside GM2, a sphingolipid, in ZIKV-infected patients relative to their non-ZIKV-infected control patients. Gangliosides, which increase membrane flexibility and are instrumental in anchoring DENV-2 replication complexes to membranes, may also anchor ZIKV replication complexes [129]. The authors suggest that since the host immune system attacks neuronal gangliosides in Guillain-Barre syndrome [130], ganglioside GM2 may be associated with this serious ZIKV complication [27]. The molecules identified in the study by Melo et al. [27,28] were not identified as being altered by infection by DENVs or CHIKV in other studies, though a direct comparison is not advisable as appropriate comparison groups for ZIKV to other flaviviruses have not yet been included in a comprehensive study.

## 5. Conclusions

The studies discussed have covered three arboviruses from the *Flaviviridae* and *Togaviridae* families and have demonstrated their influence on innumerable pathways, including the TCA cycle, fatty acid metabolism, oxidative damage, amino acid metabolism, glycolysis, immune system interactions, mTOR signaling, autophagy and others. The quantity of data gathered in just one metabolomics study is difficult to compile and understand; in reviewing the papers above, we have necessarily neglected to incorporate all information presented. This review is intended as a broad overview to guide readers to papers that will most benefit them.

Substantial gaps in our knowledge of arbovirus metabolic alterations remain. While the manipulation of host membranes by West Nile virus (WNV) has been studied in cell culture, metabolomics data from patients infected by WNV are lacking [121,122,123,131]. Arboviruses from the *Bunyavirales* order and the *Reoviridae* family also are an area that could benefit from metabolomic investigation.

### 5.1. Summary of Pathways Common among Arboviruses

Both togaviruses and flaviviruses rely on elevated glycolysis or β-oxidation of fatty acids to produce the energy they need to multiply [20,21,89,90,93,95,96,97,132]. DENVs induce autophagy and upregulate β-oxidation for energy, in the form of ATP, or for the chemical products that can be used in anabolic processes [21,89,128]. Products of β-oxidation, including acetyl-CoA, may be used to make required fatty acids and glycerophospholipids that comprise the new membranes that flaviviruses induce to house their replication complexes. Indeed, fatty acid levels are altered by flavivirus infection and inhibiting their de novo synthesis decreases production of WNV and DENVs [18,19,122]. These new fatty acids may have independent functions or may be esterified into glycerophospholipids to contribute to membrane architecture.

Membranes are important for each part of the flavivirus and togavirus lifecycles, including cell entry, replication, assembly, and exit [14,133]. Replication occurs in membrane-bound vesicles that protect vulnerable double-stranded RNA intermediates from host surveillance. Assembly of virions occurs on membranes that facilitate budding from the cell. Due to the necessity of membranes in arbovirus lifecycles, glycerophospholipids are elevated by flavivirus infection in both human and mosquito cells and generally reduced in host serum, likely due to intracellular utilization of the glycerophospholipids depleting them from the serum [11,19,20,22,88,93,97,121,123,124]. Intracellular levels of the most abundant membrane glycerophospholipid, PC, increased during infection with DENVs, WNV and ZIKV [19,82,121,124]. Flavivirus infection of humans, humanized mice, and cell culture often altered levels of glycerophospholipids that curve membranes or control membrane fluidity, such as PI, PE, LPL, and sphingolipids, consistent with the need for flexible, curved membranes for replication spherules and virion envelopes [11,20,22,27,28,85,87,93,121,123,134]. Sphingolipid and LPL production are also important for viral multiplication, as inhibiting their metabolic conversions decreased flavivirus multiplication [97,121,123].

One class of membrane-curving glycerophospholipids is LPLs, which are produced by the removal of one fatty acyl group from PC, PE, PI, PS, or phosphatidic acid. Phospholipase A2 activity, which is elevated in human serum during infection with DENVs [91], produces LPLs by cleaving *sn*-2 fatty acyls, thereby producing free fatty acids and LPLs [81,83,84]. The *sn*-2 of LPLs are enriched in unsaturated fatty acids, including AA, DHA, and ALA [81,83,84], which were elevated in the sera of patients who developed DHF, suggesting that phospholipase A2 activity is high in DHF patients [22,24]. Release of polyunsaturated fatty acids from their glycerophospholipids reservoir allows for production of pro- and anti-inflammatory signaling molecules, such as prostaglandins, leukotrienes, thromboxanes, lipoxins, and resolvins [83,84]. Hence, flavivirus production of LPLs for curved membranes may also trigger some of the inflammatory responses that characterize their infection.

Studies of arbovirus metabolomics demonstrated conserved aspects of togavirus and flavivirus manipulation of host metabolism, especially fatty acid and glycerophospholipid metabolism, but also demonstrated that unique metabolic biosignatures of different viruses make it possible to differentiate between viral infections. Alterations in human metabolism induced by infection with DENVs have been compared to changes caused by influenza virus [135], hepatitis B and hepatitis C viruses [85], and CHIKV [26].

### 5.2. Strengths of Metabolomics Analyses

Metabolomic techniques allow for sensitive and accurate measurements of many metabolites, relating to a wide variety of molecular processes simultaneously. When Birungi et al. [95] analyzed extracellular metabolites generated by DENVs in human cell culture using DIMS and ^1^H NMR, both techniques consistently identified trends in the levels of amino acids, dicarboxylic acids, fatty acids, and other TCA-related metabolites, demonstrating that different approaches to metabolomics can obtain comparable results.

A major strength of metabolomics is the ability to assess perturbations in physiological states during human infection to understand host–virus interactions in humans. Arbovirus-induced metabolic biosignatures identified in many of the studies discussed have potential for use in novel diagnostic assays [20,21,22,26,27,85,88,90]. Metabolomic methods for detecting viruses are orthogonal to both PCR and serology (Table 5). Metabolic alterations tend to occur more quickly than serologic changes and can be detected past the limited time in which viral RNA can be detected by PCR for arboviruses in serum. Metabolic changes induced by infection tend to revert to normal after the pathogen has been cleared, allowing metabolites to differentiate recent infection from previous infection with the same virus serotype. As such, metabolic assays can complement current genomic and serologic assays. Additionally, the sensitivity of metabolomic techniques allows researchers to identify metabolic changes signaling disease progression [21,22,23,24]. Serology and PCR cannot provide this prognostic data, but several metabolomics studies have identified prognostic markers of DHF [21,22,23,24]. This suggests that many of these metabolite biomarkers serve as an alarm system for specific disease states, illustrating the potential of metabolomics in better understanding these diseases. The ultimate goal of this approach is to identify the disease state within days 1–3 of fever onset and provide a path to early disease intervention, monitoring of progression or resolution, and recurrence, if any. Metabolomics data can also be used to evaluate the efficacy of therapeutic intervention by providing detailed molecular monitoring of disease states and variations from homeostasis [20].

In addition to scientific discovery, metabolomics can also be used to evaluate animal models or experimental designs. In 2017, Cui et al. [93] used LC-MS and LC-MS/MS to validate a humanized mouse model for dengue, as animal models that recapitulate dengue symptoms are lacking. The mice were extremely immunodeficient NSG mice seeded with human CD34+ fetal liver cells to produce human platelets, monocytes, and hepatocytes [136]. Cui et al. [20,93] identified many of the altered molecular features in mice to be part of the same metabolic pathways that were altered in human sera from their previous study, helping validate the humanized mice as a model for human infection with DENVs.

### 5.3. Explanation of Variation in Metabolomics Studies

Metabolomics studies can vary in many aspects that can lead to different statistically significant changes in metabolite levels. Inconsistencies between studies can arise from differences in sample collection times and preparation methods, controls, virus genotypes, host populations (including genetics and diet) or model systems, disease states, data collection methods, and analytical methods. While some groups may report significant differences in metabolite levels different from the general trends mentioned in other studies, this is not cause for alarm, but rather suggests that their data may result specifically from their sample set and analytical methods. Hence, it is difficult to build a generalizable profile of the metabolic alterations induced by any virus. For example, Cui et al. [20] and Voge et al. [22] both identified LPC(16:0), LPC(18:1), ALA, AA, and DHA as metabolites altered in human sera by infections with DENVs, but study populations from Nicaragua, Mexico, and Singapore did not show consistent trends in these metabolites. Differences in techniques, especially in chromatography (RPLC and GC versus HILIC) may be partially responsible, as well as differences in control groups (healthy versus non-dengue disease). Villamor et al. [24] noted that DHA levels were related to the DENV serotype, potentially explaining variation in DHA trends across different studies, locations, and patients. Additionally, DHA is derived from ALA, an essential omega-3 fatty acid, which is principally obtained from the diet [83]. Hence, regional and individual differences in consumption are likely.

It is important to keep in mind that different sample types reflect different aspects of metabolism. Cell lysate is distinct from cell medium. The medium is depleted or enriched with metabolites not just according to changes in metabolism, but also according to the cells’ ability to absorb, retain, or excrete metabolites. Similarly, human sera are subject to limitations of metabolite transport and may not reflect abundances of molecules within cells, which may explain why glycerophospholipids were generally elevated in cell lysate, but decreased in human sera during flavivirus infection [11,19,20,22,88,93,97,121,123,124].

Variation between studies emphasizes that metabolomic biosignatures best describe the samples used to build them, so researchers must select samples to best represent the populations and questions they seek to resolve. A standard set of methods to collect, characterize, and report samples will reduce variability between studies and improve generalizability in the same way that standardizing sample collection and processing within an experiment reduces variability. Despite different findings, the studies discussed in this review demonstrate the ability of metabolomics to differentiate disease conditions and predict disease severity. Meta-analyses of many papers or large sample sets drawn from diverse populations will likely improve the generalizability of metabolomics to clinically diagnose viral diseases.

### 5.4. Perspectives

Metabolomics provides insight into many aspects of host–virus interactions by confirming and providing more detail on previous findings, including viral energy and membrane requirements, metabolite roles in viral infection, and metabolic associations with clinical symptoms, disease states, and disease progression. The ability to broadly surveil metabolites in human sera is distinctly useful as it provides an extensive yet detailed evaluation of physiological state, and can be used to evaluate interventions during infection. Metabolomics has identified many disease biomarkers for possible future diagnostics and therapeutic treatments. However, there are several caveats and challenges to metabolomic studies [137]. There is high inter-laboratory variation in sample collection, processing and analysis. Identifying unknown metabolites and distinguishing confounding biological variations are difficult. Additionally, it is hard to obtain large independent sample cohorts that represent patient populations. Development of biosignatures is expensive and establishing the biological significance of biomarkers is challenging. Finally, when metabolomics is translated into diagnostic tests, they face slow adoption by the medical community [137]. To help overcome these challenges, appropriate control groups and well-characterized samples are essential. To minimize variability in sample collection, it is optimal to collect large numbers of prospective, rather than retrospective, samples to better control handling conditions and to account for patient variability. Miscategorized samples can invalidate results and interpretation, so the methods used to characterize samples, such as laboratory testing (PCR, serology, etc.) and collection of patient data, should be rigorous. This is important to understand if age, sex, geographic location, or other factors, such as use of specific drugs, are driving the differences observed. It is crucial to confirm metabolite identities and quantities with several data collection methods to minimize the disadvantages of individual approaches and ensure data robustness. The path from discovery of metabolites as biomarkers to an established clinical assay has been established in research on autism, newborn screening, and preeclampsia [6,138,139].

MRM assays are currently deployable in the US, as state laboratories that perform newborn screening are equipped with this technology. To expand to regions without MRM capability, alternative low-cost techniques for detecting metabolites comprising a biosignature should be developed. These may be antibody- or chemistry-based and may be ELISA-like or paper-based tests. The challenges in developing metabolism-based clinical tests for infectious diseases can be overcome by following the path set forward by other fields. Additionally, many of these metabolic pathways altered by viral infection are also altered in human disease conditions such as diabetes, metabolic syndromes, and cancers and thus have inhibitors/drugs that are already developed and on the market. Following early biomarker detection, metabolic pathways can be manipulated using already developed and tested compounds that can be re-purposed as antivirals. As illustrated in this review, metabolomics has the potential to expand our understanding of viral infections to unparalleled levels. Ongoing work combining metabolomics, genomics, and proteomics into an extensive systems biology approach will increase the strength of all three approaches, as will expanding the field of immunometabolism to link the metabolic changes discussed to immune responses involved in arboviral diseases. As technologies and metabolomic methods improve, metabolomic techniques will likely become even more valuable to increase our understanding of a multitude of diseases, syndromes, and ailments with metabolic components.

## Figures and Tables

**Table 1:** Metabolite abbreviations. Other abbreviations are listed at the end of the text.

Abbreviation	Metabolite
AA	arachidonic acid
ALA	α-linolenic acid
ATP	adenosine triphosphate
Cer	ceramide
DGLA	dihomo-γ-linolenic acid
DHA	docosahexaenoic acid
DHCer	dihydroceramide
EPA	eicosapentaenoic acid
FADH_2_	flavin adenine dinucleotide
GTP	guanosine triphosphate
LPC	lysophosphatidylcholine
LPE	lysophosphatidylethanolamine
LPI	lysophosphatidylinositol
LPL	lysophospholipid
LPS	lysophosphatidylserine
NADH	nicotinamide adenine dinucleotide
PC	phosphatidylcholine
PE	phosphatidylethanolamine
PG	phosphatidylglycerol
PI	phosphatidylinositol
PIP	phosphatidylinositol phosphate
p-PC	plasmalogen phosphatidylcholine
p-PE	plasmalogen phosphatidylethanolamine
PS	phosphatidylserine
SM	sphingomyelin
VLDL/LDL	very-low-density lipoprotein/low-density lipoprotein

**Table 2:** Selected metabolites from publications on human infections with dengue viruses (DENVs). Metabolites generally increased in disease are in red, decreased metabolites are in blue. Black metabolites have mixed results in the corresponding publication. If not specified, LC was reversed-phase. Standard single letter amino acid abbreviations are used.

Publication	Arboviruses Studied	Sample Source	Technique	Comparison	Selected Metabolites (*down in Disease*, up in Disease)	Ref. No.
Cui et al., 2013	DENV1–3 (mostly DENV1 and DENV3)	Human sera	LC-MS; GC-MS; MRM	healthy vs 3 DF time points	**fatty acids** (**AA**, ALA, **DHA**); **acylcarnitines**; glycerophospholipids (***LPC***; ***LPE***; *PC*); **glycerolipids**; **sphingolipids** (**SM**); amino acids (**F**; ***W***); **bile acids**	[20]
Voge et al., 2016	DENV2 and DENV1	Human sera	HILIC-MS; LC-MS/MS; MRM	non-dengue vs DF vs DHF/DSS	***vitamin D_3_***; glycerophospholipids (***PC***; LPC); amino acid (***P***); fatty acids (DHA; ALA; AA)	[22]
Cui et al., 2016	DENV1–4 (mostly DENV2)	Human sera	LC-MS; LC-MS/MS; MRM	DF vs DHF	***serotonin***; **kynurenine**; glycerophospholipids (PS; PE; ***LPE***)	[23]
Cui et al., 2018	DENV1–4 (mostly DENV2)	Human sera	LC-MS; LC-MS/MS	DF vs DHF	***purines***; ***acylcarnitines***; glycerophospholipids (***PC***; LPC; **LPE;*p-PC***); amino acids (**F**); fatty acids (***DHA***); **bile acids**	[25]
Khedr et al., 2015	DENV	Human blood	GC-MS	healthy vs early febrile DF	***fatty acyl esters*** (***AA***; ***DHA***)	[88]
Khedr et al., 2016	DENV	Human sera	LC-MS/MS	healthy vs DF	***glycerophospholipids*** (***LPC***; **LPI**; PC; ***PI***; *PE*; ***PS***)	[85]
El-Bacha et al., 2016	DENV3	Human sera	^1^H NMR	non-Dengue vs primary DF; secondary DF; primary DHF; secondary DHF	amino acids (A; H; *Q*; Y); ***(very) low-density lipoprotein***; carboxylic acids (acetate)	[21]
Villamor et al., 2018	DENV1–4	Human sera	GC-MS	DF vs DHF	**fatty acyl esters** (**DHA**; **AA**; **adrenic acid**; **docosapentaenoic acid**; ***DGLA***; ***pentadecanoic acid***)	[24]
Melo et al., 2018	DENV4	Human sera	DIMS; MS/MS	healthy vs DF	**glycerophospholipids (PC)**; **triacylglycerols**	[89]
Shahfiza et al., 2017	DENV	Male human urine	^1^H NMR	healthy vs DF	**hydroxy ketones**; amino acids and derivatives; ***carboxylic acids***; ** fructose**; ***myo-inositol***	[90]

**Table 3:** Selected metabolites from papers on infection with DENVs in model systems and mosquitoes. Metabolites generally increased in disease are in red, decreased metabolites are in blue. Black metabolites have mixed results in the corresponding publication. If not specified, LC was reversed-phase. Standard single letter amino acid abbreviations are used.

Publication	Arboviruses Studied	Sample Source	Technique	Comparison	Selected Metabolites (*down in Disease*, up in Disease)	Ref. No.
Cui et al., 2017	DENV2	Humanized mouse sera	HILIC and RPLC-MS; LC-MS/MS	DENV2 time points (0, 3, 7, 14, & 28 days post infection (dpi))	**fatty acids** (**DHA**, AA, ALA); **purines**; **pyrimidines**; **acylcarnitines**; **acylglycines**; ***glycerophospholipids*** (***PE***; ***PC***; *LPE*; LPC; p-PC); **sphingolipids** (**SM**); **amino acids**(**K**; ** P**); **bile acid**	[93]
Brooks et al., 1983	DENV1–4	Monkey kidney cell media	Frequency-pulsed electron-capture gas-liquid chromatography	mock vs DENV1–4	***amines***; alcohols; carboxylic acids; ***hydroxy acids***	[94]
Birungi et al., 2010	DENV1–4	human endothelial cell media	^1^H NMR; DIMS	mock vs DENV1–4 (6, 24, & 48 hpi)	**amino acids** (A; **I**; **F**; **W**; ** Y**); **keto acids**; dicarboxylic acids; fatty acids; indole acid; acyl glycine; cholesterol	[95]
Fontaine et al., 2015	DENV2	Human foreskin fibroblast cell lysate	GC-MS; LC-MS	mock vs DENV2 (10, 24, & 48 hpi)	amino acids (A; **G**; **Q**; ***W***); **carbohydrates**; glycerophospholipids (LPE; *LPC*); fatty acids (**EPA**; ** DHA**); purines; **pyrimidines kynurenine; cholesterol**	[96]
Perera et al., 2012	DENV2	C6/36 *Aedes albopictus* cell lysate	LC-MS; MRM	mock and UV-inactivated DENV2 vs DENV2	**glycerophospholipids** (**PC**; LPC; PE; **LPE**); **sphingolipids** (**SM**; **Cer**)	[19]
Chotiwan et al., 2018	DENV2	*Aedes aegypti* midguts (bloodfed)	LC-MS; LC-MS/MS; MRM	mock vs DENV2 (3, 7, & 11 dpi)	glycerophospholipids (LPC; LPE; ***LPS***; ** LPI**; **PI**; **PC**; **PE**; **PS**; PG); **glycerolipids**; **sphingolipids** (**Cer**); **fatty acyls**; ** acyl-carnitines**; sterol lipids	[97]

**Table 4:** Selected metabolites from papers on chikungunya virus (CHIKV), West Nile virus (WNV), and Zika virus (ZIKV). Metabolites generally increased in disease are in red, decreased metabolites are in blue. Black metabolites have mixed results in the corresponding publication. If not specified, LC was reversed-phase. Standard single letter amino acid abbreviations are used.

Publication	Arboviruses Studied	Sample Source	Technique	Comparison	Selected Metabolites (*down in Disease*, up in Disease)	Ref. No.
Shrinet et al., 2016	DENV and CHIKV	Human sera	^1^H NMR	Healthy vs CHIKV vs DENV vs co-infected	carbohydrates (sorbitol); amino acids (Q); pyrimidine; organic acids	[26]
Martin-Acebes et al., 2014	WNV	HeLa cell lysate	LC-MS; LC-Orbitrap	mock vs WNV	**sphingolipids** (**Cer**; DHCer; SM); **glycerophospholipids** (**PC**; **LPC**; ** p-PC**; ** p-PE**)	[121]
Merino-Ramos et al., 2016	WNV	Vero cell lysate	LC-MS	WNV infected vs WNV infected treated with ACC inhibitor	***cholesteryl esters***; sphingolipids (***Cer***; ***monohexosylCer***); glycerophospholipids (***PC***; ***PE***; *PS*) (all down in drug treated cells compared to no drug)	[122]
Liebscher et al., 2018	WNV	Vero cell lysate	LC-MS/MS	mock vs WNV	glycerophospholipids (**LPC**; PC; ***PS***; PE; PI)	[123]
Melo et al., 2016	ZIKV	C6/36 *Aedes albopictus* cells	MALDI MS; MS/MS	mock vs ZIKV infected	**sphingolipids**; **glycerophospholipids** (**LPC**; **LPS**; **PE**; **PC**); diacylglycerol	[124]
Melo et al., 2017	ZIKV	Human sera	DIMS	healthy and non-ZIKV vs ZIKV	**angiotensins**; ganglioside GM2; phosphatidylinositols	[27,28]

**Table 5:** Comparison of metabolic profiling, PCR, and serology techniques for assaying arbovirus infection.

	Metabolic Profiling	PCR	Serology
**Virus detection**	Indirect	Direct	Indirect
**Access to necessary technology**	Mass spectrometers are found in hospital laboratories (particularly those linked to universities), major clinical laboratories and laboratories performing newborn screening Not commonly found in resource-limited settings	Common to most clinical laboratories and becoming more accessible in resource-limited settings	Common to most clinical laboratories including in resource-limited settings
**Sample preparation difficulty**	Simple	Simple to difficult	Simple
**Uses**	Diagnostic and prognostic	Diagnostic	Diagnostic
**Major limitations**	Requires advanced instrumentation Currently not being applied for infectious disease diagnostics Not standardized	Laboratory contamination Dependent on viral load Specificity issues	Dependent on adaptive immune response Cannot differentiate past from current infection Cross-reactivity
**Major strengths**	Flexibility in adjusting specificity and sensitivity when monitoring multiple biomarkers Can measure rapid changes in metabolite abundance for monitoring disease progression High resolution	Rapid Highly sensitive when virus load is sufficient	Multiple platforms in diagnostic use Standardized

## Abbreviations

ACC—acetyl-CoA carboxylase; CHIKV—chikungunya virus; CoA—coenzyme A; DENV—dengue virus; DF—dengue fever; DHF/DSS—dengue hemorrhagic fever/dengue shock syndrome; DIMS—direct-injection mass spectrometry; dpi—days post infection; ELISA—enzyme-linked immunosorbent assay; GC-MS—gas chromatography–mass spectrometry; ^1^H NMR—proton nuclear magnetic resonance; HILIC—hydrophilic interaction liquid chromatography; hpi—hours post infection; LC-MS—liquid chromatography–mass spectrometry; MALDI—matrix-assisted laser desorption/ionization; MRM—multiple reaction monitoring (mass spectrometry); MS—mass spectrometry; MSI—Metabolomics Standards Initiative; MS/MS—tandem mass spectrometry; PCA—Principal Component Analysis; PCR—polymerase chain reaction; PLS-DA—partial least squares discriminant analysis; RPLC-MS—reversed-phase liquid chromatography–mass spectrometry; TCA cycle—tricarboxylic acid cycle; WNV—West Nile virus; ZIKV—Zika virus
